# Accretion of novel genetic elements reverses genome erosion and enables host-switching in the deep sea

**DOI:** 10.64898/2026.10.22.761039

**Published:** 2026-10-23

**Authors:** Camryn N Morey, Russell Corbett-Detig, Shelbi L Russell

## Abstract

Endosymbiont genome erosion is driven by relaxed selection and isolation from free-living relatives’ gene pools, and can be stalled by horizontal transmission events, but it remains unknown whether genome erosion can be reversed. The chemosynthetic gammaproteobacterial symbionts of deep-sea clams in the family Vesicomyidae offer a useful test case for whether eroded symbiont genomes can acquire novel functional content. Despite having some of the smallest genomes among marine endosymbionts, several lines of evidence, including divergent 16S rRNA variants within hosts, host-switching in sympatry, and phylogenetic signatures of repeated host transitions, suggest these symbionts retain more environmental exposure and evolutionary flexibility than expected under strict vertical transmission. Here, we use comparative genomics across the vesicomyid symbionts to test for evidence of functional gene gain. We find that three lineages have each independently expanded their genomes by approximately 25% relative to close relatives, with some expansions driven by insertion sequence (IS) or phage acquisition. The genomes that contain IS elements have disrupted genome synteny genome-wide, suggesting that these elements mediated structural rearrangements through ectopic recombination. Most strikingly, the symbiont of a thyasirid clam host, *Thyasira* sp. Haima SOB, harbors four unusually large (∼24–40 kb), highly repetitive genes with structural homology to adhesins found broadly across prokaryotes and eukaryotes. These genes show hallmarks of horizontal acquisition and IS-mediated mobilization, and their predicted export via a Type I secretion system suggests a role in mediating attachment to, or exchange of metabolites across, host cell surfaces. Considering that this symbiont lineage switched from a vesicomyid host to a thyasirid host relatively recently, and thyasirids typically host extracellular symbionts, these adhesins may have been instrumental in this host-switch. Together, these findings provide evidence that genome erosion in intracellular symbionts is not strictly unidirectional, and mobile genetic elements can serve as vectors for functional innovation, potentially enabling adaptation to new hosts and environments even after substantial genome reduction has occurred.

## Full Text Availability

The license terms selected by the author(s) for this preprint version do not permit archiving in PMC. The full text is available from the preprint server.

